# Development of Polydiphenylamine@Electrochemically Reduced Graphene Oxide Electrode for the D-Penicillamine Sensor from Human Blood Serum Samples Using Amperometry

**DOI:** 10.3390/polym15030577

**Published:** 2023-01-22

**Authors:** Deivasigamani Ranjith Kumar, Kuppusamy Rajesh, Mostafa Saad Sayed, Ahamed Milton, Jae-Jin Shim

**Affiliations:** 1 School of Chemical Engineering, Yeungnam University, 280 Daehak-ro, Gyeongsan 38541, Gyeongbuk, Republic of Korea; 2 Research Centre, Sri Sivasubramaniya Nadar College of Engineering, Tamil Nadu 603110, India; 3 Analysis and Evaluation Department, Egyptian Petroleum Research Institute, Nasr City, Cairo 11727, Egypt

**Keywords:** polydiphenylamine, electrochemically reduced graphene oxide, D-penicillamine, electropolymerization, cyclic voltammetry

## Abstract

D-penicillamine (PA) is a sulfur group-containing drug prescribed for various health issues, but overdoses have adverse effects. Therefore, regular, selective, and sensitive sensing is essential to reduce the need for further treatment. In this study, diphenylamine (DPA) was electropolymerized in an aqueous acidic medium. The PA detection sensitivity, selectivity, and limit of detection were enhanced by electropolymerizing DPA on an electrochemically reduced graphene oxide (ERGO)/glassy carbon (GC) surface. The formation of p-DPA and ERGO was investigated using various techniques. The as-prepared p-DPA@ERGO/GC revealed the excellent redox-active (N–C to N=C) sites of p-DPA. The p-DPA@ERGO/GC electrode exhibited excellent electrochemical sensing ability towards PA determination because of the presence of the –NH–functional moiety and effective interactions with the –SH group of PA. The p-DPA@ERGO/GC exhibited a high surface coverage of 9.23 × 10^−12^ mol cm^−2^. The polymer-modified p-DPA@ERGO/GC electrode revealed the amperometric determination of PA concentration from the 1.4 to 541 μM wide range and the detection limit of 0.10 μM. The real-time feasibility of the developed p-DPA@ERGO/GC electrode was tested with a realistic PA finding in human blood serum samples and yielded a good recovery of 97.5–101.0%, confirming the potential suitability in bio-clinical applications.

## 1. Introduction

**Figure 1:**
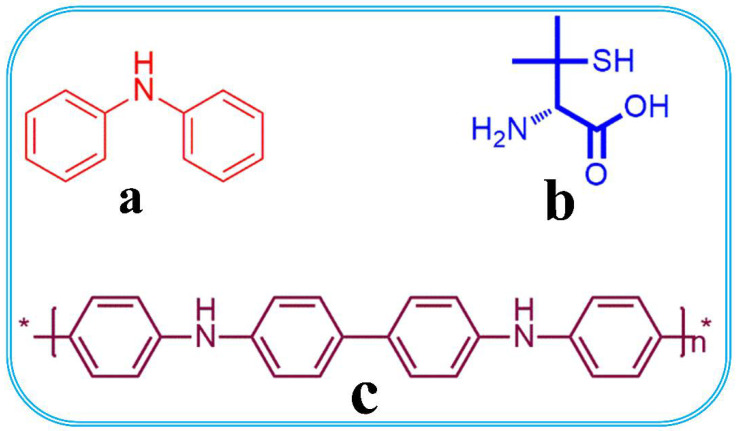
Chemical structure of (**a**) *N*,*N*-Diphenylamine; (**b**) D-Penicillamine; and (**c**) Poly(diphenylamine).

Carbon and its composites, such as carbon nanotubes/graphene, with metal nanoparticles, organic dies, and conducting polymer-based electrode materials, have been studied extensively for various analyte determinations. Carbon and its composite electrode materials showed a low limit of detection (LOD) and high sensitivity and selectivity toward the target analyte sensor [1]. Graphene-based electrode materials exhibit excellent electrochemical properties for batteries, supercapacitors, fuel cells, and electrochemical sensor applications [2]. Graphene has high conductivity and a theoretical surface area of 2630 m^2^ g^−1^, with low intrinsic mass, excellent mechanical strength, and rapid charge transport through π–electron conjugation. The reduced graphene oxide-based nanocomposites and hybrids are attractive for electrode modification [3]. Several studies have evaluated electrochemically reduced graphene oxide (ERGO)-coated electrodes for diverse applications. ERGO-modified electrodes retained some unreduced oxygen functional groups that require further surface modification, such as metal nanoparticles, metal oxides, metal sulfides, and polymers, offering excellent electrochemical performance [4,5,6]. ERGO with conducting polymers have high sensing ability because of its active redox center, rapid electron transfer rate, and high specific surface area, making them desirable for selective sensing [7]. Reduced graphene oxide surface electropolymerized with polyaniline, polypyrrole, polydopamine, polydiphenylamine (p-DPA), and polyortho-phenylenediamine revealed extraordinary electrochemical performance [7,8,9,10,11]. Among the above polymers, N,N-diphenylamine ((Figure 1), DPA)-based polymer and its composites have attracted considerable attention owing to their outstanding electrochemical redox couple activity. Obrezkov et al. used polydiphenylamine (p-DPA) as an organic cathode material for lithium and potassium-ion (dual) batteries and reported a high energy density [12]. Muthusankar et al. synthesized p-DPA with a phosphotungstic acid/graphene composite electrode electrochemically and achieved excellent selectivity towards a urea sensor [13]. Suganandam et al. synthesized a p-DPA/platinum/indium tin oxide electrode for use as an iron (III) ion sensor using spectroelectrochemical and electrochemical methods [14]. These studies emphasized that p-DPA is an auspicious electrode material for energy storage and sensor applications.

Penicillamine (PA, Cuprimine), called 2-amino-3-mercapto-3-methylbutanoic acid, is a physiological sulfur (–SH) containing amino acid (R–CH(NH_2_)COOH) in the amino-thiols family. PA exists in the L and D enantiomeric forms. L-Penicillamine is an extensive toxin that inhibits the role of pyridoxine in the body and leads to certain metabolic disorders. On the other hand, D-penicillamine (PA) is used as a therapeutic agent prescribed for treating rheumatoid arthritis, heavy metal poisoning cystinuria, liver disease, and Wilson’s disease (Figure 1) [15,16]. PA is taken directly by active rheumatoid arthritis patients in the form of an oral tablet and capsule to reduce joint pain, collagen cross-linkage, and swelling. In daily life, PA is injected at several grams per day, but only 50% of PA is consumed by the human system, which increases the PA concentration in the blood and urine [17]. An overdose of PA in human fluids may cause severe health issues, such as rashes or itchy skin, loss of taste, loss of appetite, nausea, dysentery, and serious kidney problems [18,19,20]. Despite the biological importance, the unwanted health impact of PA in pharmaceutical and biological samples needs to be determined [21].

Therefore, the researchers focused on developing simple, reliable, real-time, and accurate PA determination methods [22]. Several methods are available for PA detection, such as colorimetry [23], nuclear magnetic resonance [24], high performance liquid chromatography [25], and gas chromatography [26]. Among these methods, electrochemical methods have attracted considerable attention because of the rapid response, easy sample preparation, and no need for trained staff to operate the electrochemical device. Although the electroanalytical technique revealed user-friendly and excellent performance, it still has issues, such as narrow concentration range, high LOD, and low selectivity and electrode sensitivity. The major issue of PA determination is that the operating potential of coexisting interfering species, such as L-cysteine, uric acid, dopamine, ascorbic acid, leucine, and glucose, are close to the target analyte [27,28]. Chemically modified electrodes have resolved these problems, owing to their high conductivity, sensitivity, selectivity, rapid electron transfer rate, and low operating potential with negligible surface fouling. Modified electrodes have been designed based on nanocomposite [29], nanohybrids [30], conducting polymer [31], and metal nanoparticles [32] and applied to PA sensing in pharmaceutical and biological samples.

This paper reports the electrochemical formation of p-DPA on an ERGO/glassy carbon (GC) surface for use as an electrochemical PA sensor. The formation mechanism of the p-DPA@ERGO/GC surface involved oxidative polymerization of DPA monomer through a 4,4 coupling reaction in an aqueous acidic medium. The p-DPA@ERGO/GC electrode was used innovatively for PA determination using voltammetry and amperometric techniques. Finally, the proposed sensor examined PA in the biological human serum samples.

## 2. Experimental

### 2.1. Materials

Diphenylamine and D-penicillamine were purchased from Sigma-Aldrich (St. Louis, MO, USA). Type 1 GC plate (1 mm thickness), graphite, disodium hydrogen phosphate (Na_2_HPO_4_), monosodium hydrogen phosphate (NaH_2_PO_4_), potassium ferrocyanide (K_4_[Fe(CN)_6_], and potassium ferricyanide (K_3_[Fe(CN)_6_)] were obtained from Alfa Aesar (Shanghai, China). Potassium permanganate (KMnO_4_) and sodium nitrate (NaNO_3_) were obtained from SRL (Mumbai, India). All other regents and chemicals were of analytical grade and used as received. Deionized (DI) water was used in all solution preparations in this study. The phosphate buffer solution (PBS) pH 7.2 was prepared by mixing 0.1 M Na_2_HPO_4_ and NaH_2_PO_4_, which is used as a supporting electrolyte for PA detection.

### 2.2. Graphene Oxide Preparation

Graphene oxide was synthesized using a resemble procedure defined in former work [33]. The graphite (1 g) and NaNO_3_ (1.5 g) were placed into a one-liter beaker containing 150 mL of H_2_SO_4_ with magnetic stirring. Subsequently, 3 g KMnO_4_ was transferred carefully to the acid suspension at a reaction temperature of 5 °C. After KMnO_4_ addition, the reaction temperature was increased to 90 °C and held at that temperature for one hour, resulting in a light brownish color product. The product was diluted slowly with 300 mL of H_2_O and stirred for another two hours. Subsequently, 30 mL of 5% H_2_O_2_ were added to the above suspension, which turned brown. The product was washed with deionized water until the filtrate water was pH 7.0. The final product was freeze-dried.

### 2.3. p-DPA@ERGO/GC Electrode Assembly

**Scheme 1:**
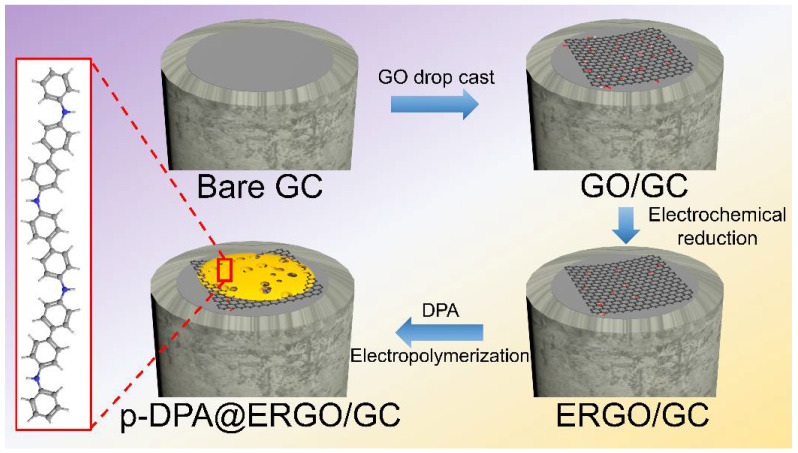
Stepwise p-DPA@ERGO/GC electrode preparation.

Scheme 1 presents the comprehensive electrode coating steps. Before the GO coating on GC, the electrode was polished with 1.0, 0.3, and 0.05-micron alpha alumina powders and swept away thoroughly with DI water. The electrode probe was then exposed to an electrochemical activation process by successive CV cycling (10 cycles) in a 0.1 M H_2_SO_4_ electrolyte in the potential window from −0.2 V to 1.0 V [34,35]. The cleaned GC electrode was used for surface modification. The as-prepared GO (5 mg) was dispersed in 5 mL of ethanol in a 5% Nafion^TM^ solution with ultrasonication for 20 min to acquire a homogenous suspension. Subsequently, 10 μL of the GO suspension spread onto a clean GC surface and dried at room temperature. The GO/GC coated electrode was subjected to electrochemical reduction in PBS (0.2 M, pH 7.2) by CV cycling over the potential range of 0.0 V to −1.5 V with fifteen sweeps to achieve the ERGO/GC. The ERGO/GC electrode underwent electropolymerization of DPA by cycling between the potential window of −0.4 V to +1.3 V in the presence of 1 mM DPA in 0.1 M H_2_SO_4_ supporting electrolyte, which yielded the p-DPA@ERGO/GC [36]. Similarly, the p-DPA@GC electrode was obtained using the same procedure on the GC surface. Similar to the above method, p-DPA@GC, ERGO/GC, and p-DPA@ERGO/GC electrodes were prepared on the GC-plate surface for X-ray photoelectron spectroscopy (XPS), Raman spectroscopy, and surface microstructure studies.

### 2.4. Instrumentation and Analytical Procedure

The surface morphology of the as-prepared GO, ERGO, and polymer film was characterized by field-emission scanning electron microscopy (FESEM, S-4800, Hitachi, Tokyo, Japan). The Raman spectra (Horiba, XploRA PLUS, Kyoto, Japan) were obtained using an operating laser wavelength of 532 nm. The formation of GO and ERGO was investigated by X-ray diffraction (XRD, PANalytical, X’Pert-PRO MPD, PANalytical, Almelo, The Netherlands) using Cu K α1 radiation (1.5406 Å). The elemental composition of the as-prepared sample was examined XPS (Thermo Scientific, Boston, MA, USA). All the electrode fabrication and PA sensor experiments (electrochemical) were performed using a PGSTAT302N electrochemical (AUTOLAB, Utrecht, The Netherlands) workstation. A 25 mL volume electrochemical cell in a three-electrode system was used for all electrochemical tests. A glassy carbon (geometry of 0.0707 cm^2^) electrode was used as the working electrode. The saturated (KCl) calomel electrode (SCE) was used as a reference electrochemical potential. A high surface geometrical area of Pt wire was used as the counter electrode. The 0.1 M PBS (pH 7.2) solution was used as a supporting electrolyte for PA determination, except for the p-DPA polymerization. A suitable volume of PA stock (0.01 M) solution was transferred to a 25 mL volume of an electrochemical cell (0.1 M PBS) in this solution immersed in three electrodes to evaluate the electrochemical results. The amperometry experiment was conducted at a constant potential of +0.62 V. In the case of cyclic voltammetry (CV) and differential pulse voltammetry (DPV) of the PA, electrochemical detection was measured over the potential of −0.20 V to +0.90 V range (0.1 M PBS).

### 2.5. Real Sample Analysis Procedure

A suitable standard aqueous PA solution was transferred to the 5% diluted human serum sample (Human serum received from Sigma-Aldrich, human male AB plasma, USA origin, sterile-filtered). The PA was determined using the standard addition method according to the amperometry technique. The PA concentration of the standard sample was yielded by extrapolating the respective standard calibration plot on the *x*-axis.

## 3. Results and Discussion

### 3.1. ERGO/GC and p-DPA@ERGO/GC Electrochemical Studies

**Figure 2:**
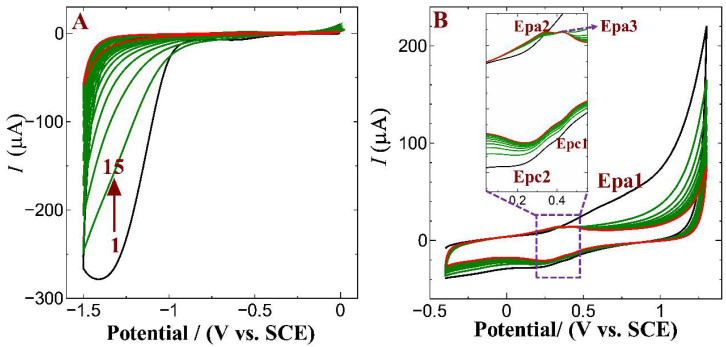
(**A**) GO electrochemical reduction CV curve trace in 0.2 M PBS (pH 7.2) at a sweep rate of 50 mV s^−1^; (**B**) electropolymerization of DPA on ERGO/GC in a 0.1 M H_2_SO_4_ contains 1 mM DPA monomer at a 50 mV s^−1^ scan rate.

Figure 2A displays the CV trace of the GO/GC electrochemical reduction process in the potential of 0.0 V to −1.50 V at a sweep rate of 50 mV s^−1^ in 0.2 M PBS (pH 7.2). The first cycle of the voltammogram showed a broad cathodic peak from −0.8 to −1.5 V. This cathodic peak corresponds to the epoxy, carbonyl, and hydroxyl group reduction [9]. The electrochemical reduction of GO to ERGO occurred by adding a proton from the supporting electrolyte and an electron from the electrode. The possible hydroxyl-group-reduction mechanism is given in Equation (1) [37,38]:

GO–OH + xH^+^ + ye^−^ → ERGO + zH_2_O (1)

In the subsequent cycles, the cathodic peak current was decreased gradually by increasing the cycle numbers, which described the removal of carbonyl, hydroxyl, and epoxy functional groups, resulting in restored sp^2^ carbon [37,38]. After 10 CV cycles, the GO/GC electrode reduction peak current reached stability, which ensures that the GO was transferred electrochemically as ERGO on the GC surface and appeared as a black color.

**Figure 3:**
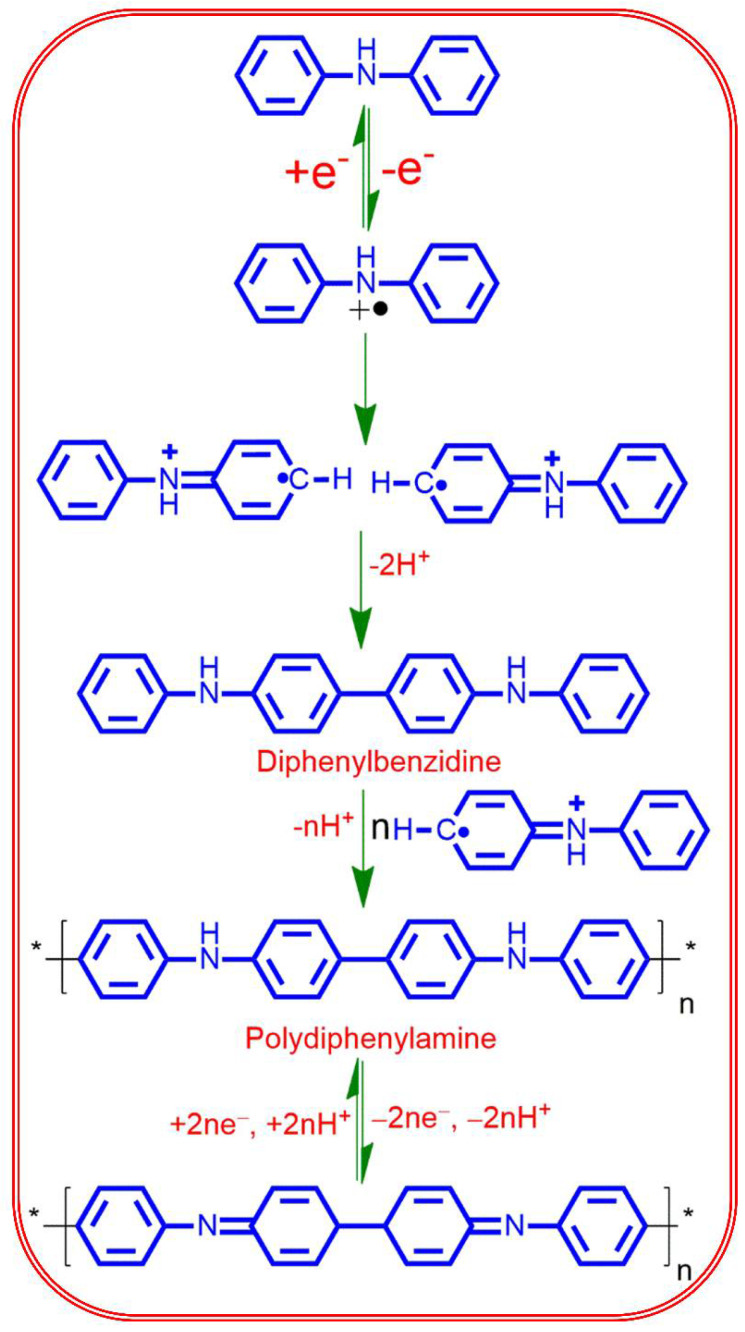
DPA stepwise electropolymerization reaction mechanism.

Figure 2B displayed a CV trace of DPA electropolymerization on the ERGO/GC electrode surface. The CV trace of 10 cycles was performed in the potential window from −0.40 V to +1.30 V in 1 mM DPA monomer with 0.1 M H_2_SO_4_ supporting electrolytes. In the first CV cycle, a broad anodic peak (Epa1) appeared at +0.55 V, corresponding to the positively charged nitrogen species of radical cation formation, followed by radical rearrangement [36]. These monomer radicals transform, dimerize, and join to form the p-DPA polymer (Figure 3). The polymer chain propagation occurred at the *para* site, such as the benzidine-type radical cation. In the reverse scan, cathodic peaks at +0.26 V to +0.4 V were observed, which correspond to the reduction of the dimeric and oligomeric products by electron/protonation. In the second cycle, the CV trace exhibited peaks at +0.32 V and +0.42 V ascribed to p-DPA oxidation and DPA monomer further deposition/conversion as a cation radical to oligomer on the ERGO/GC surface [36]. During the third and subsequent cycles, the anodic peak at +0.32/+0.23, with progressive increases in the peak current, suggests that the dimers undergo electrochemical oxidative polymerization resulting in the continued growth of p-DPA films on the ERGO/GC electrode surface. After eight cycles, the anodic (Epa2) and cathodic (Epc2) redox peak currents are stabilized, which confirmed the stable p-DPA@ERGO/GC film formation. Figure 3 shows the DPA electrochemical polymerization mechanism based on the CV trace result.

### 3.2. Structural and Surface Morphological Study

**Figure 4:**
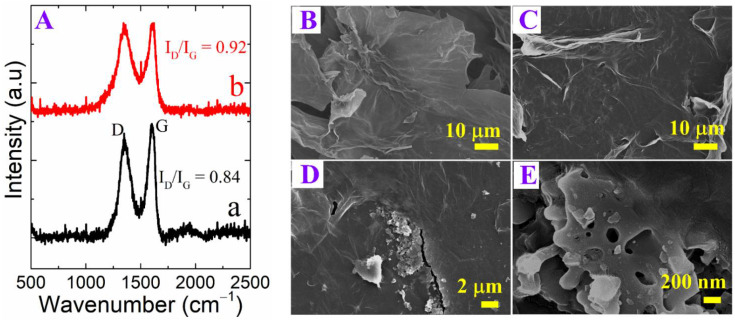
(**A**) Raman spectra of (a) GO and (b) ERGO; (**B**–**D**) FESEM of GO (**B**), ERGO (**C**), and p-DPA@ERGO (**D**); (**E**) high magnification image of p-DPA@ERGO.

The chemical functional and surface properties of the GO and ERGO were characterized by Raman spectroscopy, as shown in Figure 4(Aa,Ab). The Raman spectrum of as-produced GO exhibited two intense peaks at 1355 cm^−1^ and 1606 cm^−1^ were attributed to the D and G bands of phonons A_1g_ (K-points) and E_2g_ symmetry carbon of graphene oxide [7]. The D and G bands confirmed the chemically oxidized form of the graphene basal plane and edge defects. The D band of ERGO showed nearly the same intensity with the GO due to the increased defect level. This shows that the sp^2^ domains and edge defects confirm the electrochemical reduction of GO [39]. The calculated *I_D_*/*I_G_* ratios of GO and ERGO were 0.84 and 0.92, respectively, highlighting the significant reduction that occurred from GO to ERGO. The gained *I_D_/I_G_* value was used to calculate the average crystallite (*L_a_*) size using Equation (2) [40]:

(2) $$L_{a}\left(\mathrm{nm}\right)=\left(2.4\times 10^{-10}\right)\lambda_{l}^{4}{\left(\frac{I_{D}}{I_{G}}\right)}^{-1}$$

where *L_a_* is the average crystallite size (nm), and *λ* is the laser wavelength (532 nm) applied in the Raman spectroscopy. The calculated average crystallite size of a GO and ERGO were 22.80 and 20.80 nm, respectively. The decreasing crystallite size of GO to ERGO indicated the successful reduction of oxygen functional groups.

**Figure 5:**
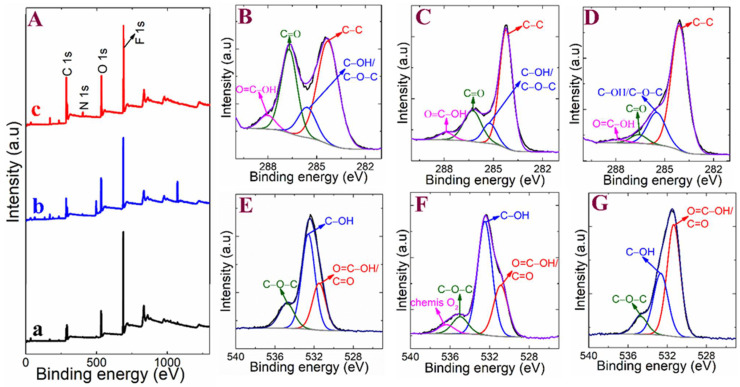
(**A**) XPS survey spectrum of (a) GO, (b) ERGO, and (c) p-DPA@ERGO; (**B**,**E**) GO C 1s and O 1s; (**C**,**F**) ERGO C 1s and O 1s; (**D**,**G**) p-DPA@ERGO/GC C 1s and O 1s region spectra.

FESEM was performed to understand the surface structure of the as-prepared GO/GC, ERGO/GC, and p-DPA@ERGO/GC film-coated electrodes (Figure 4B–E). The as-prepared GO showed a crumpled/wrinkled surface structure in Figure 4B, indicating the well-exfoliated graphene oxide layers. Figure 4C shows the oxygen functional groups removed ERGO, which revealed more crumpled/wrinkled-like surface morphology with substantial swelling. This might be due to the cathodic reduction of GO leading to the increased electrochemical active surface area or hydrogenation. The ERGO surface revealed the irregular bulk p-DPA particles, as shown in (Figure 4D,E). The amino (C-NH-C) functional group of p-DPA interacts with negatively charged (unreduced oxygen functional moiety on ERGO) groups, and the monomer aromatic π–π stacking interface is responsible for the p-DPA on the ERGO surface (Figure 5D). The high-magnified p-DPA@ERGO/GC surface morphology revealed an irregular polymer bulk particle-like structure.

### 3.3. XRD and XPS Analysis

Figure S1(Aa,Ab) (see Supplementary Materials) presents XRD patterns of the GO and ERGO on the FTO-coated electrode. The GO XRD peak appeared at 11.8° 2θ (001), indicating the 0.75 nm layer space of graphene sheets [41]. This might be because water and oxygen functional groups are dispersed within the oxidized graphene layers. After electrochemical reduction, the low angle peak diminished, and a broad shoulder peak appeared at 22° 2θ (002) explored the GO reduction, which is the removal of oxygen functional groups. The XPS spectra provide information on the sample surface elements. Figure 5(Aa–Ac) presents the GC plate surface-coated GO, ERGO, and p-DPA@ERGO samples’ survey spectra investigated results. The GO and ERGO survey traces revealed C 1s, O 1s, and F 1s peaks (source from Nafion binder). By contrast, p-DPA showed an additional N 1s peak indicating that the DPA polymerized product was on the ERGO surface. The carbon/oxygen ratio increased from GO (0.5) to ERGO (0.88), and p-DPA@ERGO (1.0) emphasized the oxygen functional group reduction through an electrochemical approach. The comprehensive understanding of functional group changes on GO, ERGO, and p-DPA@ERGO elements, the C 1s, O 1s, and N 1s, the region was deconvoluted in Figure 5B–G and Figure S1B. The high-resolution C 1s peak of GO and ERGO was fitted to the four envelopes: C–C, C–O–C/C–OH, C=O, and COOH, respectively (Figure 5B,C) [41,42]. The area under the envelope of oxygen functional groups decreased significantly, indicating the effective GO reduction (Table S1, see Supplementary Materials). Similarly, the ERGO O 1s peak area was lower than GO. Here, some of the –OH groups on the ERGO surface exploit the DPA monomer amine electrostatic interaction, leading to the facile microenvironment for polymer entrapment. In addition, p-DPA@ERGO revealed N 1s, indicating that the p-DPA polymer was successfully coated on the ERGO surface. The N 1s region is divided into two envelopes, such as imine (–N=) and amine (N–H) groups (Figure S1B, see Supplementary Materials). Table S1 (see Supplementary Materials) lists the peak positions, elements assignments, and area of the envelopes of the elements.

### 3.4. p-DPA@ERGO/GC Electrochemical Studies

**Figure 6:**
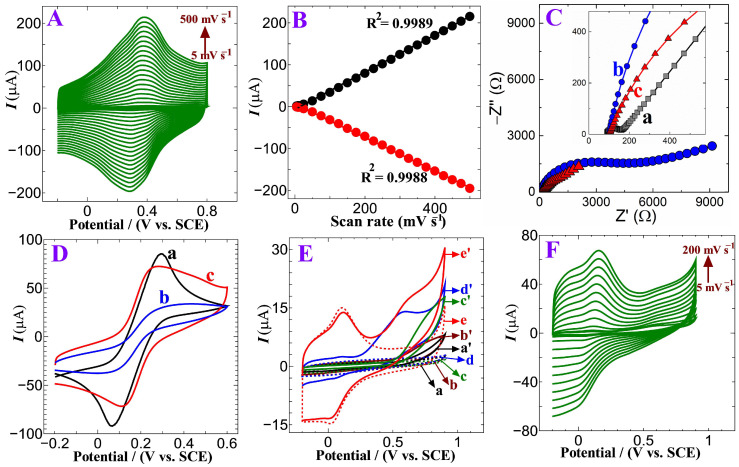
(**A**) CV scan rates of p-DPA@ERGO/GC in 0.1 M H_2_SO_4_ from 5 to 500 mV s^−1^, (**B**) corresponding plot of peak current (*I*) versus scan rate; (**C**,**D**) electrochemical impedance spectroscopy and CV of (a) bare GC, (b) p-DPA/GC, and p-DPA@ERGO/GC in [Fe(CN)_6_]^3−/4−^ (5 mM) solution of 0.1 M KCl; (**E**) CV trace of (a) bare GC, (b) activated GC, (c) p-DPA/GC, and (d) ERGO/GC, and (e) p-DPA@ERGO/GC-modified electrodes in the absence of analyte and a′, b′, c′, d′, and e′ in the presence of 1 mM PA with 0.1 M PBS at a 50 mV s^−1^ scan rate; (**F**) scan rate effect of the p-DPA@ERGO/GC electrode in PA concentration of 1 mM.

The scan rate effects were examined from 5 to 500 mV s^−1^ in the 0.1 M H_2_SO_4_ supporting electrolyte to understand the electrochemical properties of the p-DPA@ERGO/GC-coated electrode (Figure 6A). CV traces of p-DPA@ERGO/GC showed well-defined anodic and cathodic peaks, corresponding to the conversion of p-DPA and *N,N*-diphenylbenzidine protonation of the N atom in the polymer backbone [36]. The anodic/cathodic peak currents increased with increasing scan rate. As the sweep rate was increased, the anodic and cathodic peak potentials moved toward the positive and negative sides, respectively. Figure 6B presents a plot of anodic/cathodic peak current versus sweep rate, showing that the peak current increased as the scan rate was increased. This indicates the rapid electron transfer kinetics of the p-DPA film. The surface coverage (τ) of the as-prepared polymer film (p-DPA@ERGO/GC)-coated electrode was calculated from the CV curve using Equation (3) [43]:

(3) $$\tau =\frac{Q}{nFA}$$

where *Q* is a surface charge (C) obtained by integrating the CV area beneath the trace; *n* is the total number of electrons consumed in the present redox process (*n* = 2 in the p-DPA redox couple); *A* is the electrochemical surface area (p-DPA@ERGO/GC scan rate effect performed in the potassium ferrocynide solution (1 mM, in and 0.1 M KCl), using the I versus υ^1/2^ plot. The gained slope value substituted Randles–Sevcik equation for the electrochemical active surface area calculation. *A* = 0.0812 cm^2^); *F* is the Faraday constant. The calculated surface concentration of electroactive species on the electrode surface was 9.23 × 10^−12^ mol cm^−2^. The presence of electroactive p-DPA on the ERGO/GC surface enhanced surface coverage might be more feasible for determining a wide range of PA concentrations.

Electrochemical impedance spectroscopy (EIS) is a unique technique for modified electrode analysis. Table S2 (see Supplementary Materials) lists the fitted equivalent circuit model and corresponding component values. The EIS semicircle starting point of the high frequency on the *x*-axis intercept represents the electron-transfer limited process (electrolyte resistance, Rs). The Q1 and R_th_ parallel loop indicate the p-DPA and p-DPA@ERGO thin polymer materials impedance. The distance of the arch indicates the charge transfer resistance (Rct). The linear sector occurred at the low-frequency section because of a diffusion-controlled process, which dominates the electron transfer kinetics of the [Fe(CN)_6_]^3−/4−^ probe moiety on the p-DPA@ERGO/GC electrode interface [44]. Figure 6C shows the typical Nyquist plots of bare GC, p-DPA@GC, and p-DPA@ERGO/GC electrodes in [Fe(CN)_6_]^3−/4−^ (5 mM) with 0.1 M KCl medium. The bare GC and p-DPA@GC electrode revealed Rct of 156 Ω and 2790 Ω. The p-DPA@GC shows high Rct than the bare GC, which designates p-DPA alone sluggish electron transfer rate. In contrast, the p-DPA@ERGO/GC electrode revealed the Rct of 1158 Ω, suggesting effective contact between ERGO and p-DPA. The combination of ERGO and p-DPA acts as an excellent charge transfer material that allows the rapid acceleration of electrons on the [Fe(CN)_6_]^3−/4−^ redox probe with a p-DPA@ERGO/GC composite electrode.

Figure 6D shows a CV trace of bare GC, p-DPA/GC, and p-DPA@ERGO/GC-coated electrodes in the redox probe of [Fe(CN)_6_]^3−/4−^ (5 mM). The oxidation/reduction peak potential of the bare GC was observed at +0.29 V and +0.06 V, respectively. The p-DPA@GC-coated electrode showed a low peak current intensity and increased potential difference ΔEp(anodic − cathodic) = 270 mV (E_pa_ = +0.38 V; E_pc_ = +0.02 V) because of the sluggish electron transfer rate compared to the bare carbon substrate. The bulk and irregular p-DPA growth on the GC surface leads to sluggish electron transfer. In contrast, the p-DPA@ERGO/GC electrode showed a low anodic and cathodic peak potential difference ΔE_p_ = 130 mV (E_pa_ = +0.25 V; E_pc_ = +0.12 V), which is lower than the p-DPA/GC and bare GC. The higher p-DPA@ERGO/GC current than p-DPA@GC indicates that composite electrodes fast electron transfer ability. The lower ∆E_p_ values of p-DPA@ERGO/GC compared to p-DPA/GC indicated that the ERGO and polymer composite offered a high electron transfer microenvironment.

### 3.5. Electrochemical Behavior of D-Penicillamine on Modified Electrodes

**Scheme 2:**
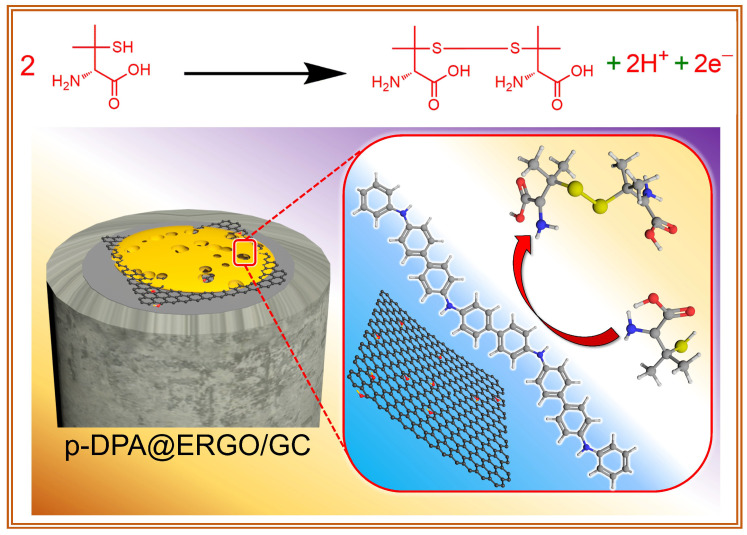
Possible electrochemical oxidation mechanism of PA at the p-DPA@ERGO/GC electrode.

The different modified electrodes were evaluated for the electrochemical detection of PA. Figure 6(Ea–Ee) represents the CV response of the bare GC, activated GC, p-DPA/GC, ERGO/GC, and p-DPA@ERGO/GC-modified electrodes absence of PA (in a 0.1 M PBS supporting medium). The CV response of bare GC, activated GC and p-DPA/GC electrodes offered a low background current in a PBS solution, but the ERGO/GC electrode showed a high background current because of its high conductivity. In contrast, the p-DPA@ERGO/GC electrode shows well-defined polymer redox couple peaks at +0.10/+0.05 V. The polymer redox potential difference (∆E_p_) for p-DPA@ERGO a 50 mV, which is lower than the p-DPA/GC-coated electrode (70 mV). This is because the combination of electrochemically active p-DPA and conductive ERGO forms an effective electroactive platform on the p-DPA@ERGO/GC electrode surface. Figure 6(Ea′–Ee′) shows CV in the presence of 1 mM PA with bare GC, activated GC, p-DPA/GC, ERGO/GC, and p-DPA@ERGO/GC-modified electrodes (0.1 M PBS supporting medium). The bare GC, activated GC, and p-DPA/GC-modified electrode showed an anodic peak at approximately +0.65 V that was related to the oxidation of PA. The ERGO/GC had a PA oxidation peak current of 13 μA. In contrast, the p-DPA@ERGO/GC electrode showed an enhanced anodic peak current of 19 μA (+0.62 V), which was approximately two times higher current and lower oxidation potential than the bare GC, activated GC, and p-DPA/GC electrode. The PA sulfuryl (–SH) functional group and polymer nitrogen group electrostatic interaction confers outstanding electrochemical performance. This leads to better PA mass transport properties. Scheme 2 shows the proposed possible mechanism of PA detection on the p-DPA@ERGO/GC electrode. Figure S2A (see Supplementary Materials) shows the different concentrations of PA (from 50 to 500 μM) addition on the p-DPA@ERGO/GC-coated electrode. The CV curve exhibited a gradual current increase as the PA concentration was increased. The corresponding calibration curve of *Ipa* = 0.0131[PA] + 3.98 with R^2^ = 0.993 (Figure S2B, see Supplementary Materials). Figure 6F presents the CV traces of various scan rate effects (5 to 200 mV s^−1^) of PA in 0.1 M PBS (pH 7.2) using a p-DPA@ERGO/GC electrode. The anodic oxidation peak current increased linearly with a positive direction potential shift as the scan rate (ν) was increased. A linear relationship existed with an anodic peak current against the square root of sweep rate (ν)^1/2^ (Figure S2C, see Supplementary Materials) with a correlation equation of *Ipa* = 2.645ν^1/2^+ 0.3480, with (R^2^ = 0.998). Furthermore, the double logarithm plot of log *i_pa_* versus log scan rate (Figure S2D, see Supplementary Materials) showed the following regression equation: log *Ipa* = 0.5148 log (ν) − 0.3867 and an R^2^ value of 0.997. These results showed that the oxidation process at the p-DPA@ERGO/GC electrode was diffusion-controlled.

### 3.6. DPV and Amperometric Detection of PA

**Figure 7:**
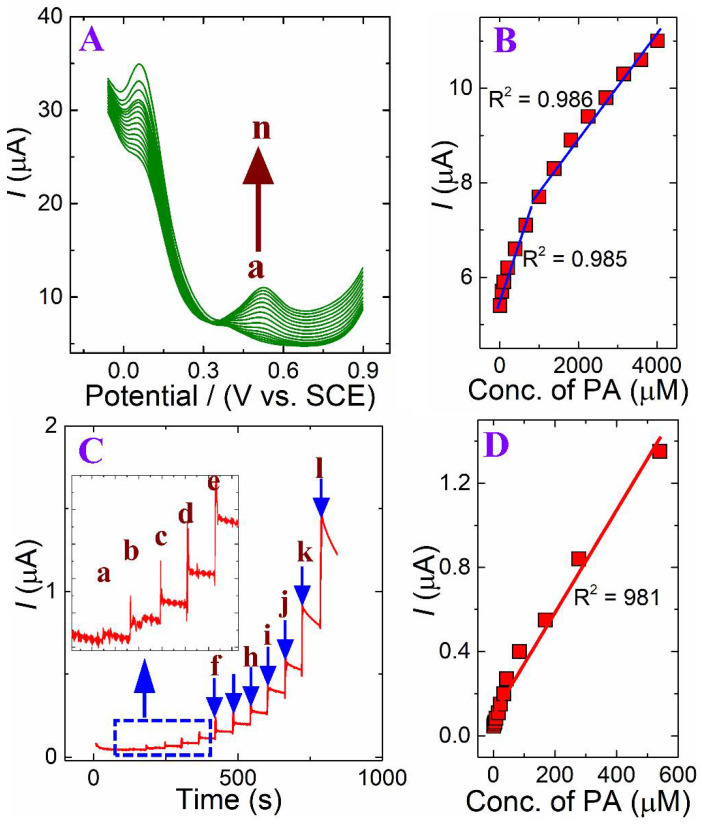
(**A**) DPV curves of different PA concentrations (a) 10.0, (b) 60.0, (c) 108.8, (d) 205.6, (e) 393.8, (f) 663.0, (g) 999.0, (h) 1386.7, (i) 810, (j) 2254.0, (k) 2706.5, (l) 3155.3, (m) 3593.8, and (n) 4015.5 μM; (**B**) calibration plot of the PA oxidation current versus concentration (pH 7.2); (**C**) amperometric (i–t) measurements for current steps upon the addition of PA from 1.4 to 541 μM on p-DPA@ERGO/GC-modified electrode with an applied potential of +0.62 V; (**D**) calibration plot of the current versus PA concentration.

The DPV technique was performed to investigate the sensitivity of the p-DPA@ERGO/GC-coated electrode towards the wide concentration of PA determination ability. Figure 7A shows the DPV of PA detection on the p-DPA@ERGO/GC electrode; the PA oxidation peak potential was observed at +0.62 V. The modified electrode sensed PA concentrations from 10 to 4015.5 µM. Figure 7B shows the equivalent calibration plot of the PA oxidation current versus concentration. The two linear regression equations for the low and high concentration ranges were *Ipa* = 0.0026[PA] + 5.593 with R^2^ = 0.985 and *Ipa* = 0.0011[PA] + 6.638 with R^2^ = 0.986, respectively. The sensitivities of low and high concentration ranges were 0.0026 µAµM^−1^ and 0.0011 µAµM^−1^, respectively. From the calibration plot, the limit of detection (LOD = 3s/S) was 0.30 µM. Figure 7C shows the successive 60 s interval PA additions performed with a fixed applied potential of +0.62 V. The amperometric current increased with every successive addition of PA with low and high concentration ranges (1.4–541 µM). The oxidation current increased considerably over a wide range as the PA concentration was increased, and Figure 7D shows the corresponding calibration curve. From the calibration curve, *Ipa* = 0.0024[PA] + 0.0984 with R^2^ = 0.981. The calculated LOD of PA on the p-DPA@ERGO/GC electrode was 0.10 µM. The linear range and low LOD values from the present study technique were compared with the existing reports (Table 1). The present p-DPA@ERGO/GC result emphasizes the developed electrode ability to determine the wide PA sensor range and low LOD ability. The obtained high sensitivity and low LOD detection ability of the developed electrode might be useful for the bio-clinical analysis of PA in blood serum samples.

### 3.7. Selectivity, Repeatability, Reproducibility, and Storage Stability

The selectivity of the modified electrode was evaluated using the amperometric technique by determining 125 μM PA in the presence of common coexisting bioactive molecules. There is a two-fold high concentration of other interfering reagents, such as ascorbic acid (AA), uric acid (UA), dopamine (DA), glucose (GLU), cysteine (CY), tyrosine (TY), and leucine (Lu). In addition, inorganic ions of Ca^2+^, Na^+^, and K^+^ were assessed (Figure S3 see Supplementary Materials). Among the foreign molecules investigated, cysteine showed a considerable influence of approximately 7% of the current responses, which can affect the determination of PA because cysteine has a similar chemical functional group. The proposed p-DPA@ERGO/GC electrode exhibits good tolerance against other common interfering bioactive molecules against PA determination. A repeatability test was performed using the same electrode set of PA detection and gained calibration plot slope values relative standard deviation (RSD, *n* = 3) to be 3.2%. The reproducibility of the developed sensor electrode was also studied in three replicate studies of the p-DPA@ERGO/GC-modified electrode, which was evaluated in 1 mM PA and resulted in an RSD (*n* = 3) of 3.85%. In addition, storage stability performed with a set of DPV measurements was carried with the p-DPA@ERGO/GC electrode after 20 days stored at 5 °C and obtained a similar current response for the determination of PA (RSD = 4.8). The observed selectivity, repeatability, reproducibility, and storage stability showed that the p-DPA@ERGO/GC-modified electrode is a potential tool for realistic PA detection.

### 3.8. Bio-Analytical Applications of p-DPA@ERGO/GC Electrode

The practical bio-analytical applicability of the as-prepared electrode was analyzed using real human serum samples. The determination of PA in serum samples performed by the standard addition method, and the obtained results are given in Figure S4A–D (see Supplementary Materials) and Table S3 (see Supplementary Materials). The results showed good recoveries of PA: 97.5 and 101.0%, with RSD values of 6.0 and 6.5%, respectively. These results confirmed that the p-DPA@ERGO/GC could be applied to the real-time analysis of PA in biological samples.

## 4. Conclusions

A p-DPA@ERGO/GC electrode was developed as a PA sensor. The detailed electrode fabrication through electrochemical reduction of GO and electropolymerization diphenylamine on the ERGO surface were discussed. The as-fabricated electrode was investigated by Raman spectroscopy, FESEM, EIS, and CV. The p-DPA formation mechanism on ERGO was given. The p-DPA@ERGO/GC-modified electrode was used for the oxidative determination of PA. The sulphuryl functional group of PA can adsorb on the electroactive amino functional surface of the p-DPA@ERGO/GC electrode. Amperometry revealed PA detection over a wide concentration range (1.4–541 µM), as well as high sensitivity, selectivity, and a low detection limit (100 nM). In addition, the designed sensor electrode was used in the real-time determination of PA in human serum samples, highlighting its bio-clinical applications.

## Figures and Tables

**Table 1:** p-DPA@ERGO/GC electrode performance on PA detection compared to previous reports.

Electrode	Method	Linear Range (µM)	Limit of Detection (µM)	Ref.
Cu^2+^/carbon paste electrode	CV	1–1000	0.1	[45]
β-CD@Ca-sacc/MeOH/GC ^a^	DPV	100–500	0.79	[46]
CeO–ZnO/ILFCPE ^b^	DPV	0.02–25.0	0.01	[47]
KI/GC	DPV	9.0–120	3.5	[48]
AuNPs/RGO ^c^	DPV	5.0–110	3.9	[49]
t-Butylcatecho/GC	Amperometry	0.02–80	0.007	[50]
Quinizarine/TiO_2_/CPE	SWV ^d^	0.8–140	0.76	[51]
p-DPA@ERGO/GC	Amperometry	1.4–541	0.10	**This work**

^a^ β-cyclodextrin/self-assembled chiral skeleton; ^b^ ionic liquid/ferrocene derivative n-hexyl-3-methylimidazolium hexafluoro phosphate; ^c^ reduced graphene oxide; ^d^ square wave voltammetry.

## Data Availability

The data presented in this study are available in the lab research notebook in SSN College and Yeungnam University.

## Supplementary Materials

The following are available online at https://www.mdpi.com/article/10.3390/polym15030577/s1. Figure S1. (A,B) CV of concentration of PA addition and calibration plot, (C) plot of PA anodic oxidation peak current versus square root of scan rate, (D) double logarithmic plot of peak current versus scan rate; Figure S2. Amperometric selectivity curve; Figure S3. DPV standard addition human serum sample analysis; Figure S4. (A,C) Amperometry current response to the diluted human serum sample, by standard addition (R+S) method (B,D) corresponding analytical curve of the standard addition; Table S1. XPS region deconvolution evelopes peak positions, assignment, FWHM, and area; Table S2. The modified electrodes EIS data Randles circuit fitted values; Table S3. D-penicillamine determination in the human serum samples using p-DPA@ERGO/GC electrode.
