# Supplementary material for: Development of Polydiphenylamine@Electrochemically Reduced Graphene Oxide Electrode for the D-Penicillamine Sensor from Human Blood Serum Samples Using Amperometry
Source: Polymers (Basel). 2023 Jan 22;15(3):577. doi: 10.3390/polym15030577 (PMC9921737; doi:10.3390/polym15030577)
Supplement: Supplementary file 1 [file polymers-15-00577-s001.zip › polymers-2098726-supplementary.pdf]

polymers

MDPI

Article

Development of Polydiphenylamine@Electrochemically Reduced Graphene Oxide Electrode for the D-Penicillamine Sensor from Human Blood Serum Samples Using Amperometry

Deivasigamani Ranjith Kumar [1,*], Kuppusamy Rajesh [2,*], Mostafa Saad Sayed [1,3], Ahamed Milton [1] and Jae-Jin Shim [1,*]

[1] School of Chemical Engineering, Yeungnam University, 280 Daehak-ro, Gyeongsan 38541, Gyeongbuk, Republic of Korea; mostafassayed@gmail.com (M.S.S.); miltonahamedru@gmail.com (A.M.)
[2] Research Centre, Sri Sivasubramaniya Nadar College of Engineering, 603110 Tamil Nadu, India
[3] Analysis and Evaluation Department, Egyptian Petroleum Research Institute, Nasr City, Cairo 11727, Egypt
* Correspondence: ranjith@yu.ac.kr (D.R.K.); rajeshche05@gmail.com (K.R.); jjshim@yu.ac.kr (J.-J.S.)

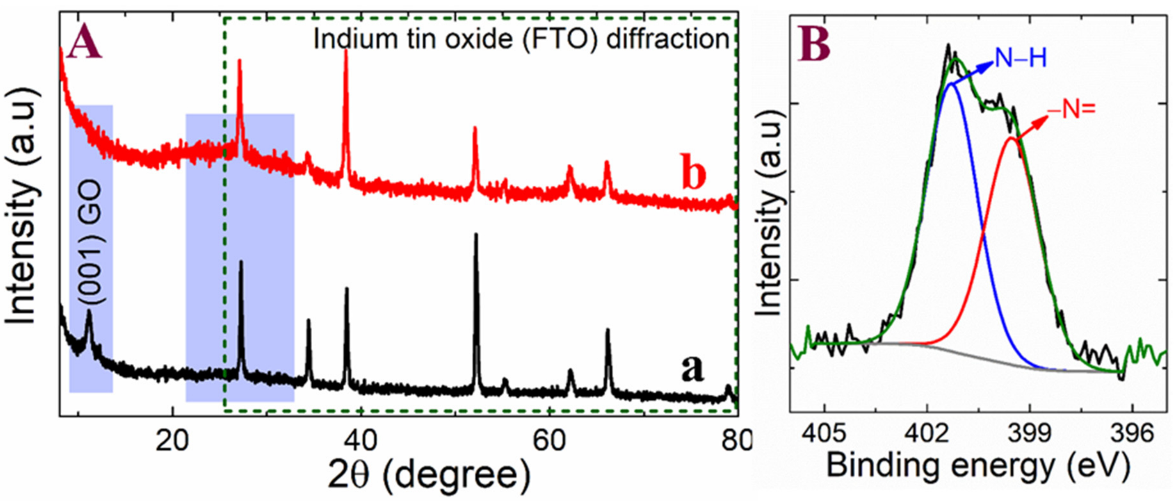

Figure S1. (A) XRD pattern of (a) GO/FTO and (b) ERGO/FTO, (B) XPS N 1s region spectrum.

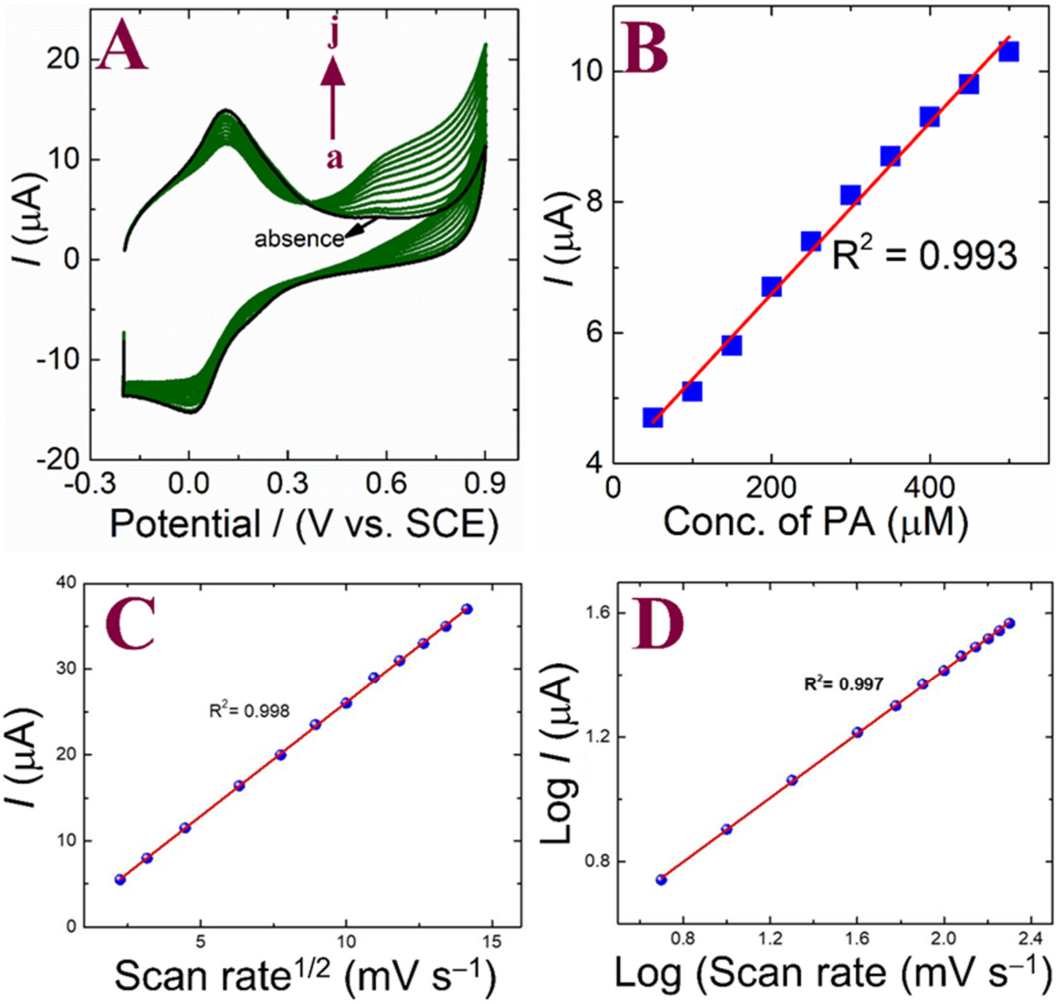

Figure S2. (A) CV trace of p-DPA@ERGO/GC with different concentrations of PA addition from 50 to 500 μM, (B) corresponding peak current versus PA concentration, (C) Plot of PA anodic oxidation currents versus square root of scan rate, (D) double logarithmic plot of peak current versus scan rate.

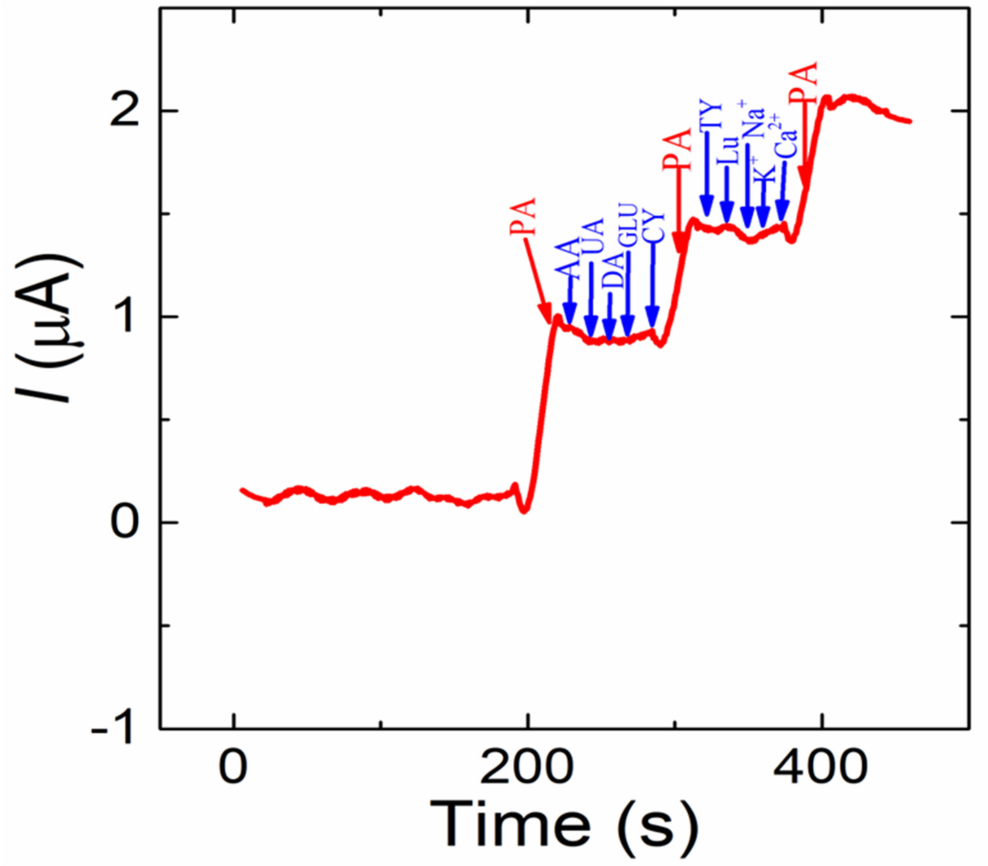

Figure S3. The amperometric curve of PA detection (125 µM) with the two-fold higher concentration of other common interfering agent AA, UA, DA, GLU, CY, TY, Lu, Na^+, K^+, and Ca^{2+}.

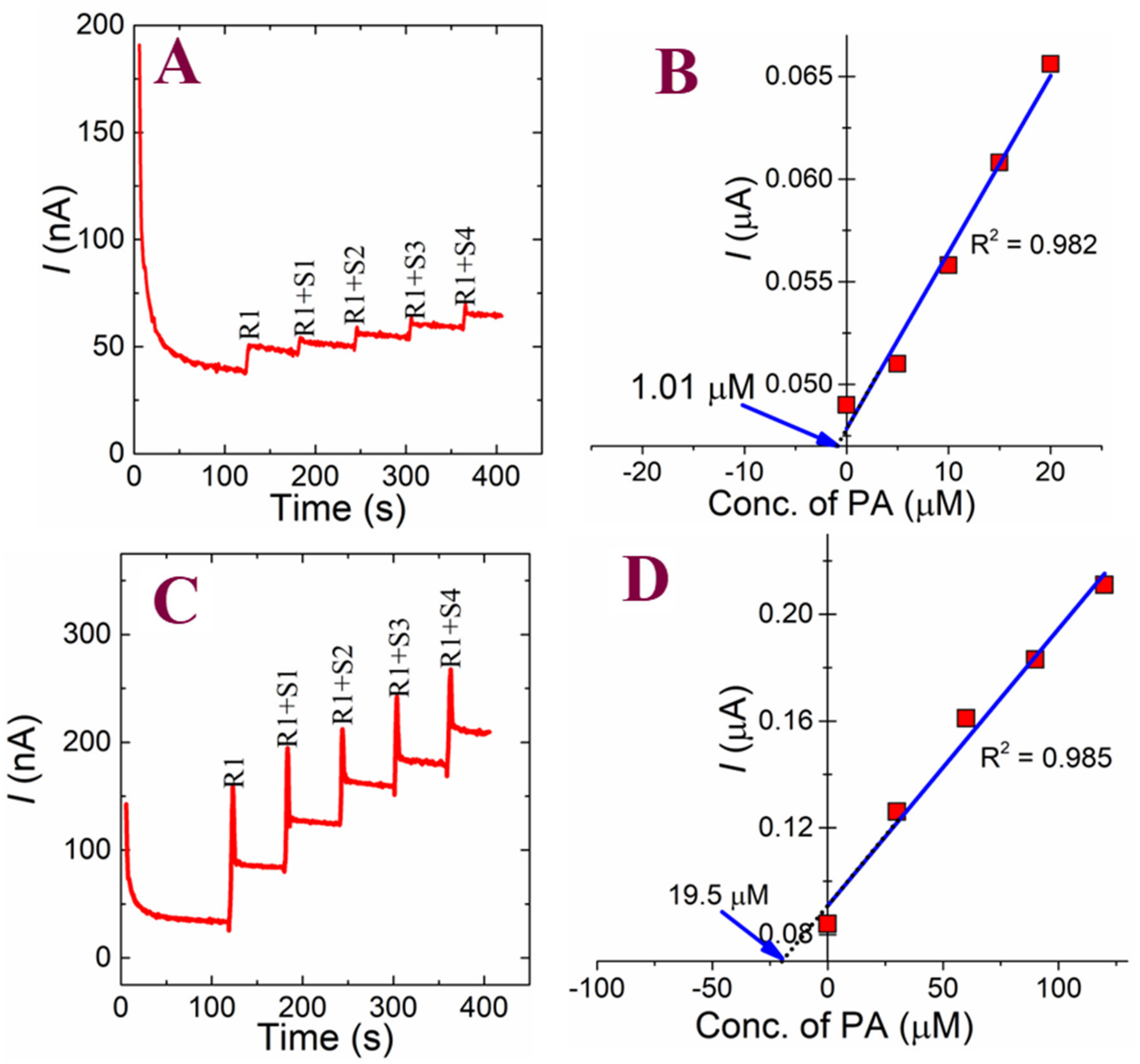

Figure S4. (A and C) Amperometry current response to the diluted human serum sample, by standard addition (R+S) method (B and D) corresponding analytical curve of the standard addition.

Table S1. XPS region deconvolution evelopes peak positions, assignment, FWHM, and area.

Electrode	Element	Binding energy	Assignment	FWHM	Area
GO/GC	C 1s	284.4	C–C	1.5	15701
		285.6	C–OH/C–O–C	1.3	3925
		286.7	C=O	1.2	9563
		288.0	O=C–OH	1.1	1573
	O 1s	531.5	O=C–OH/C=O	1.6	19480
		532.6	C–OH	1.6	28961
		534.8	C–O–C	1.8	8688
ERGO/GC	C 1s	284.5	C–C	0.94	19587
		285.3	C–OH/C–O–C	0.99	3525
		286.3	C=O	1.2	6901
		288.0	O=C–OH	1.0	1380

	O 1s	531.0	O=C-OH/C=O	1.7	18226
		532.5	C–OH	1.7	40503
		534.9	C–O–C	1.8	6730
		536.3	Chemisorbed O_2	1.8	3540
**p-DPA@ERGO/GC**	C 1s	284.5	C–C	1.8	31293
		285.5	C–OH/C–O–C	1.3	8918
		286.6	C=O	1.2	2190
		288.0	O=C–OH	1.4	938
	O 1s	531.3	O=C–OH/C=O	1.6	29110
		532.6	C–OH	1.7	17466
		534.7	C–O–C	1.7	4946
	N 1s	399.5	–N=	1.8	2590
		401.3	N–H	1.7	2911

Table S2. The modified electrodes EIS data Randles circuit fitted values. .

**Electrode**	**R_s (ohm/cm^2)**	**Q_1 (Fs$^{(a-1)}$)**	**a_1**	**R_{th} (ohm/cm^2)**	**Q_2 (Fs$^{(a-1)}$)**	**a_2**	**Rct (ohm/cm^2)**	**W (ohm s$^{-1/2}$)**	**Equivalent circuit**
Bare GC	85	6.50×10^{-6}	0.74				156	7066	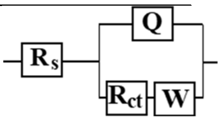
p-DPA/GC	99	2.40 ×10^{-6}	0.86	184264	0.0015	0.20	2790		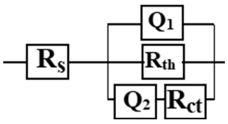
p-DPA@ERGO/GC	101	0.78 ×10^{-3}	0.72	5625	0.0014	0.79	1158		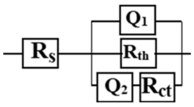

Table S3. D-penicillamine determination in the human serum samples using p-DPA@ERGO/GC electrode.

**Sample no**	**Added (μM)**	**Found (μM)**	**Recovery (%)**
Human serum 1	1	1.01	101.0
Human serum 2	20	19.5	97.5

RSD values for the human serum sample detections (n=3) are less than 6.0 and 6.5 %.
